# Early detection of metastatic uveal melanoma by the analysis of tumor‐specific mutations in cell‐free plasma DNA

**DOI:** 10.1002/cam4.4153

**Published:** 2021-07-21

**Authors:** Claudia H. D. Le Guin, Norbert Bornfeld, Nikolaos E. Bechrakis, Leyla Jabbarli, Heike Richly, Dietmar R. Lohmann, Michael Zeschnigk

**Affiliations:** ^1^ Department of Ophthalmology University Hospital Essen University Duisburg‐Essen Essen Germany; ^2^ Department of Medical Oncology West German Cancer Center University Duisburg‐Essen Essen Germany; ^3^ Institute of Human Genetics University Hospital Essen University Duisburg‐Essen Essen Germany

**Keywords:** cell‐free DNA, cell‐free tumor DNA, deep amplicon sequencing, GNAQ/GNA11, oncogenic mutations, uveal melanoma

## Abstract

**Background:**

Eye salvaging therapy of malignant melanomas of the uvea can preserve the eye in most cases, but still about half of patients die from metastatic disease. Previous analyses of cell‐free DNA from plasma had shown detectable levels of tumor‐specific *GNAQ*/*GNA11* mutations in patients with the clinical diagnosis of progressive disease. However, data on the **time** span that elapses from the detection of ctDNA in plasma to the clinical detection of metastases (diagnostic lead time) are missing.

**Methods:**

We examined 135 patients with uveal melanoma. Cell‐free DNA was isolated from a total of 807 blood samples which were taken over a period of up to 41 months and analyzed for the presence of *GNAQ*/*GNA11* mutations by deep amplicon sequencing.

**Results:**

Twenty‐one of the 135 patients developed metastases or recurrence. A ctDNA signal was identified in the plasma of 17 of the 21 patients. In 10 patients, this ctDNA signal preceded the clinical diagnosis of metastasis by 2–10 months. In 10 other patients, a ctDNA signal was only detected in samples obtained shortly before or after radiotherapy. The presence of a ctDNA signal in 16 of the remaining 125 patients was linked to clinical manifestation of metastases (*n* = 14) or tumor recurrence (*n* = 2) with a sensitivity and specificity of 80% and 96%, respectively.

**Conclusion:**

Detection of ctDNA in plasma can provide a diagnostic lead time over the clinical diagnosis of metastases or tumor recurrence. Longer lead times are to be expected if intervals between sampling are shortened.

## Graphical abstract

In metastasized uveal melanoma (UM) patients, circulating tumor DNA (ctDNA) can be detected in blood. Here we explored if cfDNA is a suitable biomarker for the early detection of metastatic disease in UM patients. Our data show that this biomarker fulfills the expectation as, overall, about half of the patients who developed metastases showed a positive ctDNA signal prior to the clinical diagnosis of metastatic disease with a lead time ranging between 2 and 10 months. Moreover, it is reasonable that, with more frequent sampling time points, diagnostic lead times will be even longer.

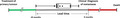

## 1 INTRODUCTION

Uveal melanoma (UM) is a malignant intraocular tumor. Incidence in Europe and the United States is between 2 and 8 cases per 1 million.^1^, ^2^ Although current therapies achieve satisfactory local disease control, about half of the patients die of metastatic disease. The most common site of metastasis is the liver (90%), followed by lung (24%) and bone (16%).^3^ Less frequently, other organs such as skin or kidney are affected by metastasis.^4^

Two major classes of UM have been recognized which are associated with patients’ prognosis.^5^, ^6^, ^7^ Thus, molecular classification of primary UM is often used to predict the patient's risk of metastatic disease.^8^, ^9^ In some cases, surgical resection of metastases might improve the survival of the patient^10^ but it must be noted that effective therapies for patients with metastatic disease for wide application are still missing. Currently, different therapeutic approaches are being tested, with immunotherapeutic strategies being considered as a possible option.^11^ It is assumed that chances of successful treatment of metastatic disease in both the metastatic and adjuvant setting are better in patients with lower tumor burden.^12^ Therefore, it is plausible that the prognosis of patients will improve by the early detection and treatment of the metastases.

Current monitoring strategies for the early detection of metastases after the successful treatment of primary UM rely on liver function test (LFT) combined with liver imaging by ultrasound/MRI or computed tomography. The sensitivity of the LFT, that is, the fraction positive LFT findings in patients with metastatic disease, is rather low at 24% if the monitoring period was limited to 90 days before the diagnosis of metastasis.^13^ In addition, serum markers such as lactate dehydrogenase and alkaline phosphatase are used to estimate tumor load in clinical routine.^14^

A possible alternative to serum markers for monitoring progressive disease is the analysis of circulating cell‐free (cfDNA) in the plasma of patients.^15^ A high level of cfDNA can provide information for the early detection of metastases and recurrent disease.^16^ In patients with solid tumors, cfDNA in the blood originates from tumor cells and from normal cells. Although higher rates of apoptosis and necrosis in tumors compared to normal tissues increase the probability of tumor DNA release, the cfDNA from normal cells usually outweighs the cell‐free tumor DNA (ctDNA).^17^ The availability of assays based on detection of tumor‐specific mutations allows to distinguish ctDNA from DNA of normal cells and facilitates unambiguous detection and quantification of tumor cell‐derived DNA in the plasma of patients.^18^

Up to 92.5% of UMs show oncogenic somatic mutations at either position Q209 or R183 of one of the two paralogue genes GNAQ or GNA11.^19^, ^20^, ^21^ GNAQ mutations affecting position Q209 are also found in circumscribed choroidal hemangiomas. However, the genetic variants present in this benign neoplasia are distinct from that in UM as they result in the replacement of glutamine by arginine (Q209R). Therefore, we can assume that all GNAQ/11 alleles with UM‐specific variants detected in the cfDNA are derived from UM DNA.^22^, ^23^ Thus, these mutations qualify for the unequivocal detection of tumor DNA in the plasma of patients.

In a previous study, it was shown that UM‐specific mutations and thus ctDNA can be identified in the plasma of patients with metastatic UM.^24^ Here we used this assay to conduct a prospective study in 135 consecutive UM patients in order to obtain data on the temporal relationship between ctDNA detection in plasma and clinically detectable tumor progression.

## 2 MATERIALS AND METHODS

### 2.1 Patients and study design

Between October 2014 and October 2016, we asked 226 patients to participate in the study. As the availability of primary tumor tissue is a prerequisite, only patients who either received an enucleation, transretinal endoresection after neoadjuvant single‐dose gamma knife irradiation, or tumor biopsy (transretinal or transscleral) prior to brachytherapy were included. Material from transretinal or transscleral tumor biopsy was available from patients who decided for prognostic testing. Of the 151 patients who met these criteria, 135 patients had an oncogenic *GNAQ* or *GNA11* mutation in their tumor.

Blood withdrawal was scheduled prior to and 1 day after primary tumor sampling in all patients included in the study. Over a period of up to 41 months following tumor sampling, blood samples were obtained whenever the patient presented at our department for tumor control. The follow‐up time is defined here as the time interval between the first blood sampling and the last time that information was available. Patients were screened every 3 months by their local oncologist for the presence of metastases.

Written informed consent was given by every patient included in this study and the Declaration of Helsinki protocols have been followed. This study has been approved by the Ethics committee of the University Duisburg‐Essen.

### 2.2 Plasma preparation and cfDNA isolation

Blood was taken by venipuncture, the plasma was prepared, and cfDNA extracted from plasma as previously described.^24^

### 2.3 Sequence analysis

Sequencing of DNA from primary tumor samples and deep amplicon sequencing of the cell‐free DNA was performed as described previously.^23^ Paired‐end sequencing on the Illumina MiSeq platform resulted in a median read depths of 77,769 reads per sample.

### 2.4 Statistics

Data analysis was performed with JMP11 (SAS) and the R software (R version 3.6.0, https://www.R‐project.org) using packages bundled in the tidyverse (Welcome to the tidyverse. Journal of Open Source Software, 4(43), 1686, https://doi.org/10.21105/joss.01686).

## 3 RESULTS

### 3.1 Clinical characteristics

**FIGURE 1:**
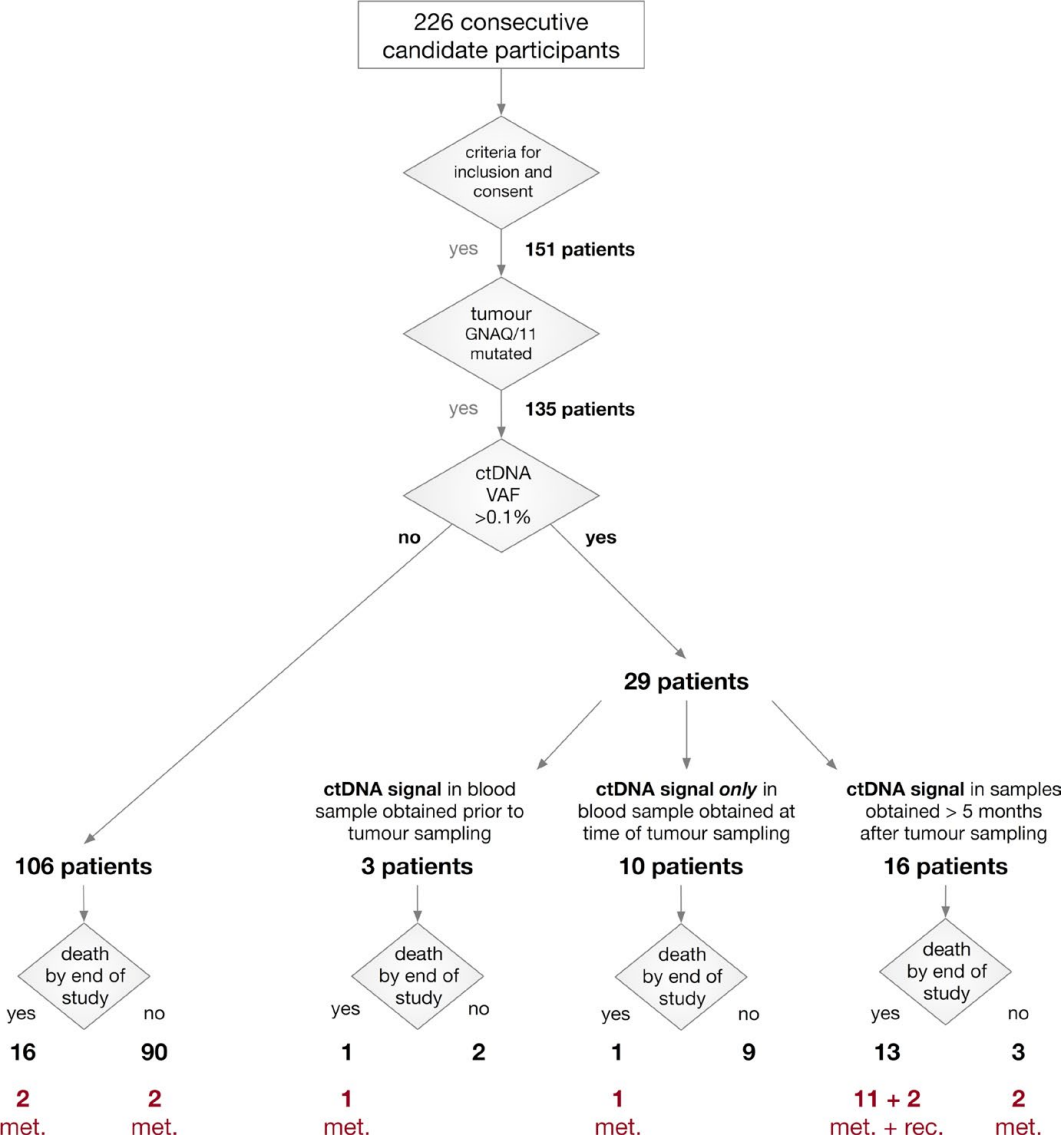
Overview of the study cohort and the grouping of patients depending on the absence or presence of ctDNA (values above the level of detection, VAF = 0.1%) and survival. In the group of 106 patients without ctDNA increase, 16 patients died. Two of them died from UM metastases and the cause of death is unknown in 14 of these patients. In those two patients with intraocular or extraocular tumor recurrence, the cause of death is also unknown. All other deceased patients died from UM metastases. Met, metastasized patients. Rec, patients with extra‐ or intraocular recurrence; UM, uveal melanoma; VAF, variant allele fraction

Among 226 consecutive patients who were eligible to participate in the study, 151 patients gave informed consent. From all these patients, tumor tissue for *GNAQ*/*11* genotyping was available (Figure 1). In 135 of these patients, an oncogenic *GNAQ* or *GNA11* variant, which allows unequivocal detection of ctDNA in the plasma, was found in the tumor (Table S1). From each of these 135 patients, blood was withdrawn before and at various time points after tumor sampling. Thus, a total of 807 blood samples were collected, the cfDNA was isolated and analyzed for the presence of *GNAQ*/*GNA11* mutations using deep‐amplicon sequencing as described previously.

Median age at diagnosis of all patients was 61 years and the median tumor height and median largest basal diameter was 5.5 and 10.9 mm, respectively. Ciliary body involvement was present in 14 (10%) and extraocular tumor growth was detected in 2 (1.5%) patients. Monosomy 3 was identified in 61 (45%) tumors. The remaining 74 tumors showed either disomy 3 or partial monosomy 3 (Table S1).

**FIGURE 2:**
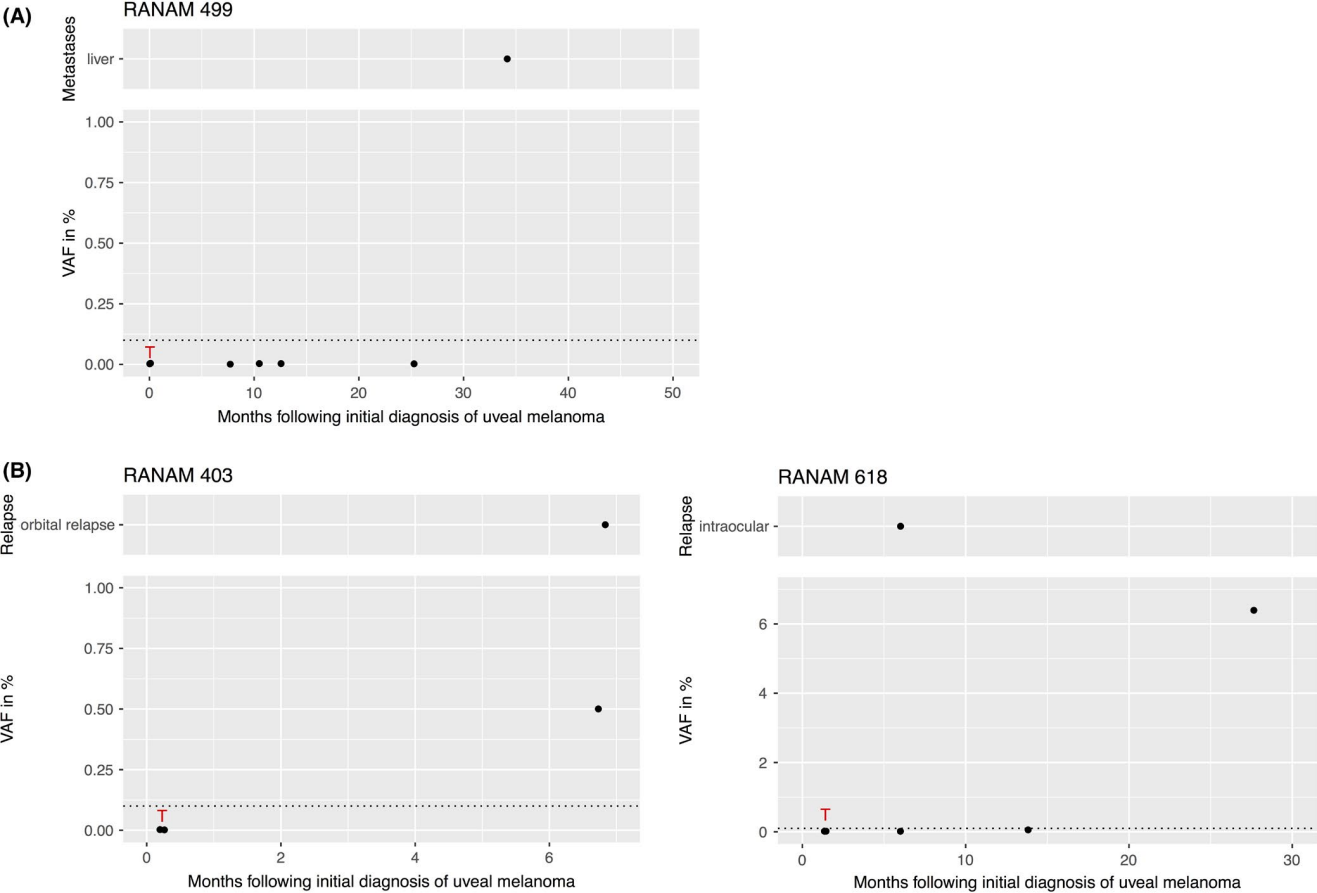
VAFs of mutant *GNAQ* or *GNA11* alleles in UM patients at different time points after the initial diagnosis of the primary tumor (Lower part). (A) Example of a metastatic patient who did not show an increase in ctDNA until the end of the study. (B) Two patients with intra‐ or extraocular recurrence. Red T: Time point of sampling of tumor tissue. Dotted line: level of detection at VAF = 0.1%. Upper part: location and time of clinical diagnosis of the recurrence (RAN403 after 6.8 months and RAN618 after 7 months). UM, uveal melanoma; VAF, variant allele fraction

The median follow‐up time was 46 months (range 8–64 months). During follow‐up, one patient presented with local extraocular tumor recurrence and another patient presented with local intraocular recurrence (Figure 2B). A total of 31 patients died by the end of the study and in 16 of these patients, including both patients with local recurrence, we were unable to obtain information on the cause of death or possible metastasis. The remaining 15 patients had metastases diagnosed clinically. Another four patients developed metastases but were still alive at the end of the study. In 18 of 19 patients who developed metastatic disease, the liver was the primary site of metastasis and in the remaining patient, metastases were first discovered in the bones.

### 3.2 Genetic analyses

Sanger sequencing of *GNAQ*/*GNA11* in DNA from primary tumors revealed oncogenic mutations at either position Q209 or R183 in 135 patients (Table S1). From these 135 patients, 807 blood samples (range 2–9 blood samples per patient) were collected during the study period. The cell‐free DNA was isolated from all samples and analyzed for the presence of the *GNAQ*/*GNA11* mutation, previously found in the matched primary tumor, by deep amplicon sequencing. This method allows the determination of the variant allele fraction (VAF) as a measure of the proportion of ctDNA in the total cfDNA.^23^ We considered a plasma sample ctDNA positive if the mutant *GNAQ* or *GNA11* alleles showed a VAF >0.1%.

#### 3.2.1 Patients without ctDNA signal at any sampling time point

In 106 of the 135 patients, the VAF of the tumor‐specific mutation in cfDNA did not exceed the detection limit of 0.1% at any time (Figure 1). Of these 106 patients, 16 died until the end of the study. Clinically diagnosed metastatic disease was the cause of death in two of them. No information about the cause of death was available in the remaining 14 patients. Of the 106 ctDNA negative patients, 90 were still alive at the end of the study and two of them developed clinically diagnosed metastases during follow‐up (RANAM499 [Figure 2A], RANAM659). To our knowledge, four patients developed metastases without a corresponding increase in ctDNA.

#### 3.2.2 Patients with positive ctDNA signal only prior or soon after tumor sampling

**FIGURE 3:**
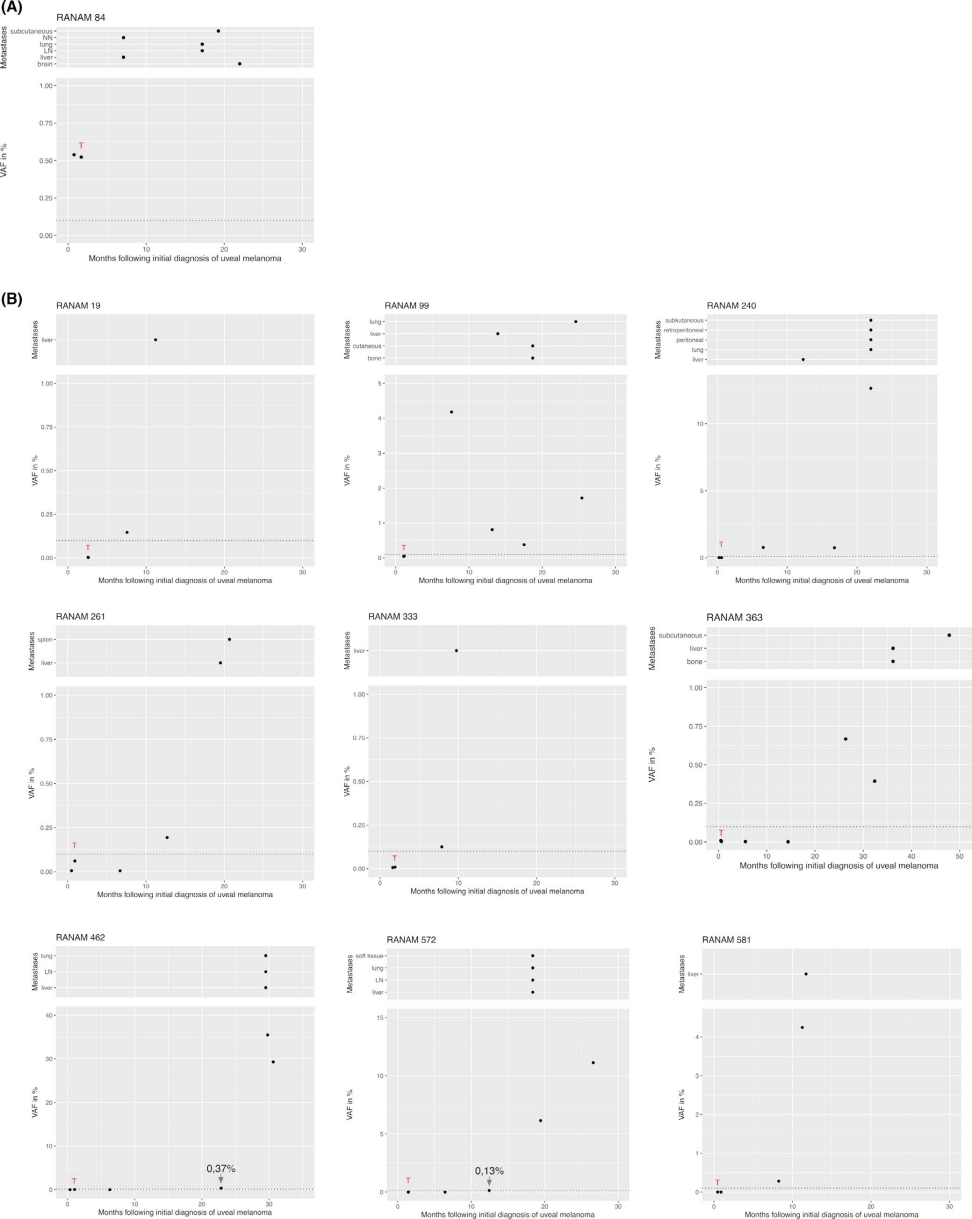
VAF of mutant *GNAQ* or *GNA11* alleles in plasma with a ctDNA signal preceding the clinical diagnosis of metastasis. One patient with a positive ctDNA signal at the time of therapy (A) and nine patients with ctDNA increase >5 months after therapy (B). Legend descriptions see also Figure 2. Upper part: location and time of clinical diagnosis of the metastases. LN, lymph node; NN, adrenal cortex; VAF, variant allele fraction

In three of the 135 patients, a ctDNA signal was present in the blood sample obtained prior to tumor sampling (Figure 1). One of these patients (RAN0084, Figure 3A) was clinically diagnosed with metastases 5 months later, which we interpret as the detection of a ctDNA signal in this patient 5 months prior to the clinical diagnosis of metastatic disease.

In 10 patients, ctDNA was only detected in blood samples taken at the time of tumor sampling. In three of them, a transretinal tumor biopsy was performed including one who received a transscleral tumor biopsy subsequently followed by brachytherapy with a bi‐nuclide (ruthenium 106/iodine 125) plaque under which the blood sample was taken. The remaining 7 of the 10 patients were treated with neoadjuvant single dose gamma‐knife irradiation followed by transretinal endoresection and brachytherapy with ruthenium‐106. In one of the patients treated by endoresection, ctDNA was detected at the same time when the clinical diagnosis of metastases was made. In summary, in seven of the nine non‐metastatic patients with a positive ctDNA signal present at the time of tumor sampling only, this blood sample was collected immediately after or during tumor therapy (gamma knife irradiation followed by endoresection with adjuvant brachytherapy or brachytherapy). Since none of these seven patients showed an increase in ctDNA or metastasis in the further course of the study, the temporal correlation indicates that the detected tumor DNA was released in the context of the therapy.

#### 3.2.3 Patients with a positive ctDNA signal more than 5 months after tumor sampling

Sixteen patients showed ctDNA in blood samples taken at least 5 months after the sampling of the primary tumor tissue (Figure 1). Of these, 13 (81%) were dead by the end of the study and in 11 of them, metastatic disease was known to be the cause of death. The two other patients with positive ctDNA signals had developed tumor recurrences, one intraocular and the other extraocular (Figure 2B). We could not establish the cause of death of these patients. Three of the 16 patients were alive by the end of the study, two of them with the diagnosis of metastatic disease. One of the three surviving patients showed a ctDNA signal in the sample collected 23 months before the end of the study. Whether or not he developed metastases remains unknown.

#### 3.2.4 cfDNA signals in the patients with the clinical diagnosis of metastatic disease

A total of 19 patients developed metastases and two patients local recurrences. In two of the 19 patients, the ctDNA‐positive samples were obtained at the time of tumor therapy (Figure S1). Monosomy 3 was found in 18 of the 21 tumors (85%) with positive ctDNA signal and the average AJCC score of 2.9 was higher in the ctDNA positive tumors than in the group of tumors (average AJCC = 2.0) in which no ctDNA was detected. The enrichment of tumors with monosomy 3 in the group of ctDNA positive patients is to be expected, as this tumor class has a significantly increased metastatic potential.

**FIGURE 4:**
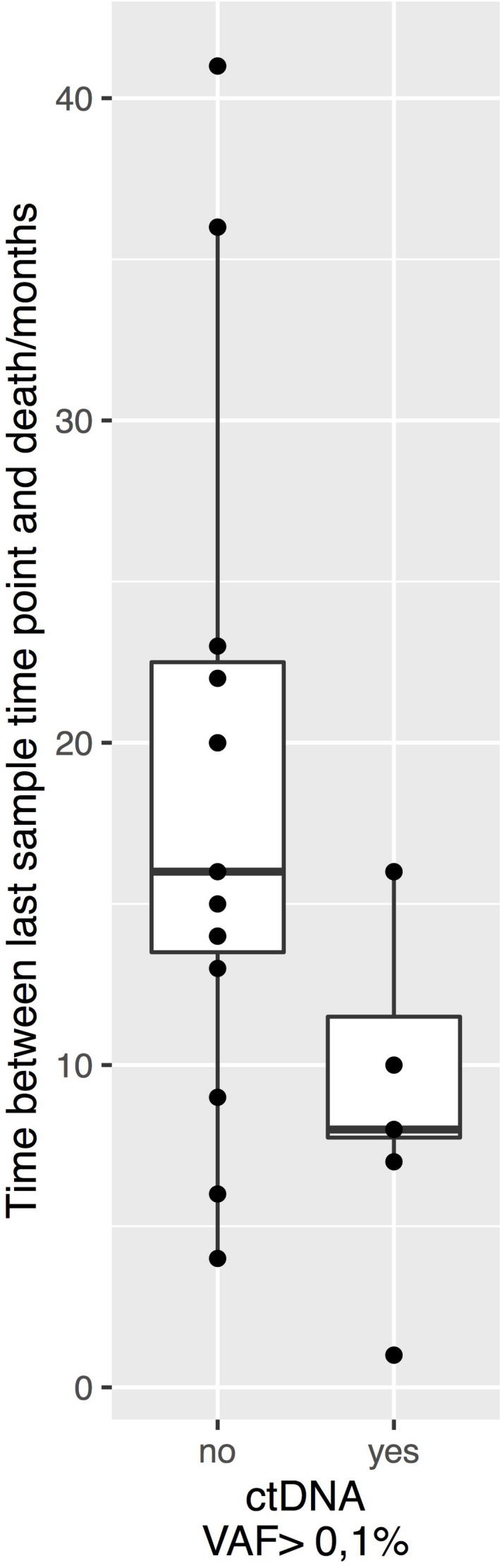
Time interval between the last blood sampling and the death of the patients in the 14 ctDNA negative patients (no) and in the eight ctDNA positive patients (yes) in which ctDNA was detected prior to the clinical diagnosis of metastases. In the ctDNA positive patients, we used the time point when the first ctDNA positive sampling was taken. ctDNA, circulating tumor DNA

In 15 of the 21 patients, a positive ctDNA signal was detected in samples obtained >5 months after therapy. Nine of these patients showed the presence of ctDNA in samples that were taken prior to clinical diagnosis of metastases (Figure 3B). The mean time period between obtaining the first sample with a positive ctDNA signal and diagnosis of metastases was 5.7 months (range 2–10 months). Eight of these nine patients died before the end of the study with the median period of 8 months elapsed between the first ctDNA positive samples until death (Figure 4).

In four of the 21 patients with metastases or local recurrences, no positive ctDNA signal was detected at any time point. One of these patients left the study early and was thus not available for regular blood sampling. In the three remaining patients without ctDNA signal, the time interval between the last sample collection and the clinical diagnosis of metastases was 9 months (RANAM3, 17 months; RANAM499 [Figure 2A], 9 months and RANAM659, 17 months).

## 4 DISCUSSION

In this prospective study, we have collected 807 blood samples from a cohort of 135 consecutive UM patients at different time points relative to the sampling of the primary tumor tissue. Tumor‐specific *GNAQ*/*GNA11* mutations in the cell‐free DNA were determined using deep amplicon sequencing and the proportion of mutant versus wild‐type sequences was used as a measure for the proportion of tumor‐derived DNA in the plasma of the patients.

### 4.1 Positive ctDNA signals are infrequent prior or soon after tumor sampling

In only 3 of the 135 patients, a ctDNA signal was present in the blood sample obtained prior to tumor sampling. In one of these patients, (RANAM84) metastatic progression was diagnosed 5 months later. Conversely, most patients (132 of 135) with intraocular UM did not show ctDNA signal prior to treatment of the primary tumor. Thus, it appears that at this stage of disease, tumor DNA released from untreated intraocular UM, if present, is below the limit of our detection method.

In this context, it is worth mentioning that our measurement of ctDNA in peripheral blood at a certain point in time provides a snapshot of a dynamic equilibrium that is influenced by the release and clearance of tumor DNA. The clearance rates are variable, which is indicated by different half‐lives of the cfDNA depending on the given physiological conditions.^25^, ^26^ Furthermore, the clearance rate may also depend on the cfDNA concentration itself in a nonlinear way.^27^ The absence or low concentrations of ctDNA prior to tumor treatment likely reflects a lower rate of tumor cell death, moreover, the amount of ctDNA in the bloodstream may also depend on the permeability of the blood–brain barrier, as assumed by Khier and Lohan.^28^ Since under brachytherapy of the primary tumor, that is, a few days after endoresection, an increase of ctDNA in the blood is observed in some patients, it is obvious that such a barrier cannot fully block the release of ctDNA into the bloodstream.

We found ctDNA in blood samples obtained at the time of tumor sampling in seven patients who did not, until the end of our study, develop clinically detectable metastases. In six of these patients, blood samples were obtained while the patients were under brachytherapy. None of these patients was ctDNA positive at time points >5 months after tumor sampling. It remains to be shown whether the increase in ctDNA in these UM patients is a direct response to radiotherapy as has already been shown for patients with other tumor types.^29^ For UM patients, upcoming studies will investigate whether such a connection exists and whether radiation therapy is causally involved.

### 4.2 Specificity and sensitivity of ctDNA testing for the detection of metastatic disease

The main goal of this prospective study was to evaluate if ctDNA signals precede clinical diagnosis of metastases during the follow‐up of patients with UM. We set the start of the observation period at 5 months after therapy because we assumed that any ctDNA signals after this time interval could not originate from the primary tumor but must originate from metastases or tumor recurrence.

**FIGURE 5:**
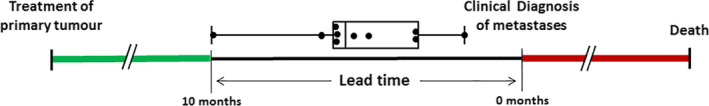
Distribution of lead times of the detection of progressive disease by cfDNA testing prior to the clinical diagnosis of metastases in nine UM patients (black dots). cfDNA, circulating cell‐free DNA; UM, uveal melanoma

During the follow‐up stage, 18 patients presented with metastasizing disease >5 months after tumor sampling, one patient at the time of tumor sampling. In two further patients, extra‐ or intraocular local tumor recurrences were detected. A ctDNA signal was detected in 16 of these patients. Based on these data, the estimated sensitivity (true positive rate) of ctDNA detection for detecting metastases or tumor recurrence is 80% (16/20). In the group of ctDNA negative patients, 14 patients died with unknown cause of death. It is plausible that at least some of these ctDNA negative patients have died from metastasizing disease. Assuming, that all of these 14 patients had metastases then the estimated sensitivity of our ctDNA biomarker test would be 47% (16 out of 34) (Table S2). The second relevant metric for the assessment of test performance is its specificity, the true negative rate. Until the end of the study, 105 patients remained free of clinically detected metastases or relapse. A ctDNA signal was detected in four of these patients. From this figure, the specificity is estimated at 96% (101/105) (Table S2). Specificity remains high even if assuming that all patients who were dead by end of the study died of metastasizing UM. In addition to sensitivity and specificity, the potential clinical utility of this ctDNA biomarker test also depends on diagnostic lead time. The ctDNA signal preceded the clinical manifestation of metastases in 9 of the 20 patients with metastases (Figure 3B). In addition, one of the three patients (RANAM84) with a positive ctDNA signal prior to tumor sampling had a clinical diagnosis of metastatic disease 5 months later (Figure 3A). Thus, 10 of 20 (50%) patients with metastases or recurrence had a positive ctDNA biomarker test prior to the clinical diagnosis of disease progression with diagnostic lead times ranging between 2 and 10 months (mean 5.7 months) (Figure 5). This compares well to lead times observed in other tumors including breast cancer^30^ and urothelial bladder cancer.^31^

The ctDNA biomarker test was false‐negative (1‐ true positive rate) in four patients. Although it is reasonable to assume that the test may produce false‐negative results in some patients, none of the four cases in our study is well suited to support this assumption. One patient left the study early, thus preventing early ctDNA detection. In the three remaining patients without ctDNA signal, the time period from last blood sampling and clinical diagnosis of metastases was longer than 9 months. In comparison, the diagnostic lead time in patients with ctDNA signal prior to clinical diagnosis was between 2 and 10 months. These data reflect the kinetics of metastatic progression and the rise of VAFs to the levels above the limit of detection of the biomarker test. Thus, to improve the sensitivity of the test, it appears that the time intervals between blood sampling must be shortened. Another area of improvement is the ease of use of the preanalytical phase. It is easier for patients to participate if blood samples can be drawn at their place of residence and shipped at ambient temperature. This can be achieved by the use of special blood collection tubes designed for the stabilization of cell‐free DNA and limited release of genomic DNA for several days at room temperature.^32^

## 5 CONCLUSIONS

In metastasized UM patients, circulating tumor DNA (ctDNA) can be detected in blood. Here we explored if cfDNA is a suitable biomarker for the early detection of metastatic disease in UM patients. Our data show that this biomarker fulfills the expectation as, overall, about half of the patients who developed metastases showed a positive ctDNA signal prior to the clinical diagnosis of metastatic disease with a lead time ranging between 2 and 10 months. Moreover, it is reasonable that, with more frequent sampling time points, diagnostic lead times will be even longer.

## CONFLICTS OF INTEREST

The authors declare no conflict of interest.

## ETHICAL APPROVAL

Written informed consent was given by every patient included in this study and the Declaration of Helsinki protocols have been followed. This study has been approved by the Ethics committee of the University of Duisburg‐Essen (Approval ID 13–5462‐B0).

## Supporting information

Figure S1

Table S1

Table S2

## Data Availability

The data that support the findings of this study are available from the corresponding author upon reasonable request.
