# Supplementary figures and images for: Early detection of metastatic uveal melanoma by the analysis of tumor‐specific mutations in cell‐free plasma DNA
Source: Cancer Med. 2021 Jul 21;10(17):5974–82. doi: 10.1002/cam4.4153 (PMC8419753; doi:10.1002/cam4.4153)

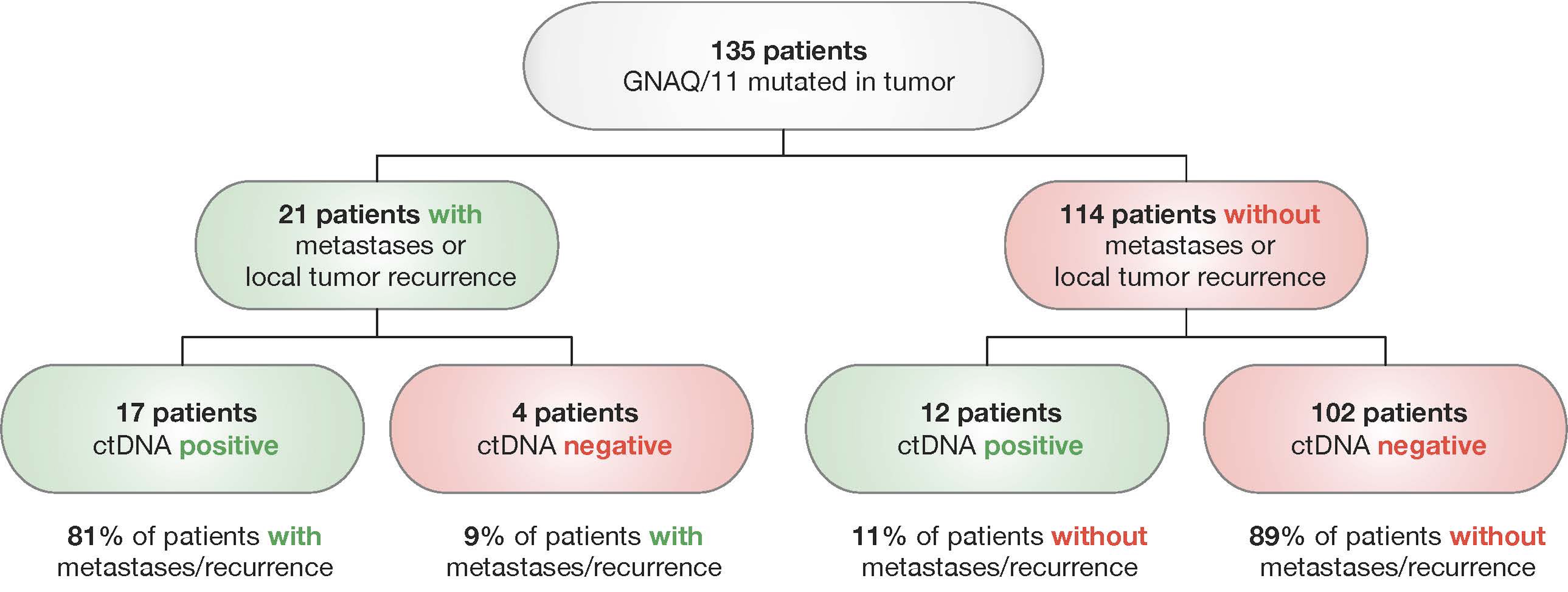

Supplement: Supplementary file 1 — Figure S1 [file CAM4-10-5974-s003.jpg]
