# Supplementary material for: Early detection of metastatic uveal melanoma by the analysis of tumor‐specific mutations in cell‐free plasma DNA
Source: Cancer Med. 2021 Jul 21;10(17):5974–82. doi: 10.1002/cam4.4153 (PMC8419753; doi:10.1002/cam4.4153)
Supplement: Supplementary file 2 — Table S1 [file CAM4-10-5974-s002.docx]

**Supplemental Table 1:**

| Clinical features |  |
| --- | --- |
| Median follow-up time (months) | 46 (range 8 – 64) |
| Median Age at diagnosis (years) | 61 (range 34-82) |
| Median tumour height (mm) | 5.5 (range 1 – 17.8) |
| Median LBD (mm) | 10.9 (range 2.6 – 22) |
| Ciliary body involvement n (%) | 14 (10%) |
| Extraocular tumour growth n(%) | 2 (1.5%) |
| Genetic status: n (%)  M3  D3 and partial M3 | 61 (45%)  74 (55%) |
| 8^th^ AJCC classification n (%) |  |
| I | 50 (37%) |
| II | 29 (21.5%) |
| III | 42 (31.1%) |
| IV | 14 (10.4%) |
| GNAQ mutations (n) |  |
| GNAQ Q209 | 60 (44.4%) |
| GNAQ R183 | 1 (0.7%) |
| GNA11 Q209 | 67 (49.6%) |
| GNA11 R183 | 7 (5.2%) |

**Supplemental Table 1:** Clinical characteristics and GNAQ/GNA11 mutation type of 135 patients
