# Supplementary material for: Early detection of metastatic uveal melanoma by the analysis of tumor‐specific mutations in cell‐free plasma DNA
Source: Cancer Med. 2021 Jul 21;10(17):5974–82. doi: 10.1002/cam4.4153 (PMC8419753; doi:10.1002/cam4.4153)
Supplement: Supplementary file 3 — Table S2 [file CAM4-10-5974-s001.docx]

| **Supplemental Table 2**  **Detection of metastases or recurrence**   \|  \| metastazised or recurrence \| metastases and recurrence free \| \| --- \| --- \| --- \| \| ctDNA positive \| 16 \| 4 \| \| ctDNA negative \| 4 \| 101 \| \| all \| 20 \| 105 \| \| sensitivity \| 0.8 \|  \| \| specificity \|  \| 0.96 \| | | |
| --- | --- | --- | --- | --- | --- | --- | --- | --- | --- | --- | --- | --- | --- | --- | --- | --- | --- | --- | --- | --- |
| **Detection of metastases or recurrence assuming that all deaths died from metastases** |  |  |
| \|  \| metastazised, recurrence or dead \| metastases and recurrence free or alive \| \| --- \| --- \| --- \| \| ctDNA positive \| 16 \| 3 \| \| ctDNA negative \| 18 \| 88 \| \| all \| 34 \| 91 \| \| sensitivity \| 0.47 \|  \| \| specificity \|  \| 0.97 \| |  |  |
